# Clinicians’ perceptions of usefulness of the PubMed4Hh mobile device application for clinical decision making at the point of care: a pilot study

**DOI:** 10.1186/s12911-018-0607-9

**Published:** 2018-05-08

**Authors:** Kyungsook Gartrell, Caitlin W. Brennan, Gwenyth R. Wallen, Fang Liu, Karen G. Smith, Paul Fontelo

**Affiliations:** 1 Department of Nursing, Towson University, Linthicum Hall Room 201J, 8000 York Road, Towson, MD 21252 USA; 2 National Institutes of Health Clinical Center Nursing Department, 10 Center Drive, Bldg. 10/6-3523, Bethesda, MD 20892-1151 USA; 3 National Institutes of Health Clinical Center Nursing Department, 10 Center Drive, 6-1484, Bethesda, MD 20892 USA; 4 National Library of Medicine, Lister Hill National Center for Biomedical Communications, B1N30N, 38A, 8600 Rockville Pike, Bethesda, MD 20894 USA; 5 National Institutes of Health/Library, 10 Center Drive, Bethesda, MD 20892 USA; 6 National Library of Medicine, Lister Hill National Center for Biomedical Communications, B1N30L, 38A, 8600 Rockville Pike, Bethesda, MD 20894 USA

**Keywords:** Mobile application, Clinical decision making, Point of care, PubMed4Hh

## Abstract

**Background:**

Although evidence-based practice in healthcare has been facilitated by Internet access through wireless mobile devices, research on the effectiveness of clinical decision support for clinicians at the point of care is lacking. This study examined how evidence as abstracts and the bottom-line summaries, accessed with PubMed4Hh mobile devices, affected clinicians’ decision making at the point of care.

**Methods:**

Three iterative steps were taken to evaluate the usefulness of PubMed4Hh tools at the NIH Clinical Center. First, feasibility testing was conducted using data collected from a librarian. Next, usability testing was carried out by a postdoctoral research fellow shadowing clinicians during rounds for one month in the inpatient setting. Then, a pilot study was conducted from February, 2016 to January, 2017, with clinicians using a mobile version of PubMed4Hh. Invitations were sent via e-mail lists to clinicians (physicians, physician assistants and nurse practitioners) along with periodic reminders. Participants rated the usefulness of retrieved bottom-line summaries and abstracts and indicated their usefulness on a 7-point Likert scale. They also indicated location of use (office, rounds, etc.).

**Results:**

Of the 166 responses collected in the feasibility phase, more than half of questions (57%, *n* = 94) were answerable by both the librarian using various resources and by the postdoctoral research fellow using PubMed4Hh. Sixty-six questions were collected during usability testing. More than half of questions (60.6%) were related to information about medication or treatment, while 21% were questions regarding diagnosis, and 12% were specific to disease entities. During the pilot study, participants reviewed 34 abstracts and 40 bottom-line summaries. The abstracts’ usefulness mean scores were higher (95% CI [6.12, 6.64) than the scores of the bottom-line summaries (95% CI [5.25, 6.10]). The most frequent reason given was that it confirmed current or tentative diagnostic or treatment plan. The bottom-line summaries were used more in the office (79.3%), and abstracts were used more at point of care (51.9%).

**Conclusions:**

Clinicians reported that retrieving relevant health information from biomedical literature using the PubMed4Hh was useful at the point of care and in the office.

**Electronic supplementary material:**

The online version of this article (10.1186/s12911-018-0607-9) contains supplementary material, which is available to authorized users.

## Background

The ability to use information-seeking behavior with specific clinical questions, methods, and reliable websites are important to clinicians [1, 2]. A survey of clinicians by White et al. [3] found that only 65% used PubMed often. Although they were aware of PubMed website, more than half (59%) of clinicians had no formal training on how to use it. Findings from a study of online access to MEDLINE in clinical settings showed that in 60% of cases, clinical decisions were made using titles, medical subject headings, and abstracts [4]. Furthermore, physicians reported that journal abstracts and full-text articles equally increased the accuracy of clinical decisions in clinical simulations [5]. Meanwhile, there is little information available regarding how clinicians use evidence from research abstracts to make clinical decisions [6]. Wireless mobile devices can alleviate barriers to access and improve delivery of reliable clinical information to support clinical decision making at the point of care. Fontelo and colleagues [1] noted that clinicians using a Web version of PubMed4Hh [PubMed for Handhelds] preferred “bottom-line summaries” more than abstracts. The PubMed4Hh mobile application can be useful for providing clinicians with a quick way to access online biomedical literature in support of evidence-based medicine at the point of care if it is integrated into clinical workflow.

Sackett provides the classic definition of evidence-based medicine as, “*the conscientious, explicit, and judicious use of current best evidence in making decisions about the care of individual patients*” [7]. Evidence-based medicine (EBM) as described in this definition used scientific evidence and technology to determine best practices in the 1980s. Sackett and Straus [8] used an ‘Evidence Cart’ for physicians in order to provide easy access to evidence for clinical decisions during rounds. The ‘Evidence Cart’, which physicians carried during their clinical rounds, contained multiple sources of printed materials, such as evidence from Cochrane Library, MEDLINE, a physician examination textbook, a radiology anatomy textbook, and a Simulscope. Nowadays, the bulky ‘Evidence Cart’ has been replaced by the “virtual evidence cart” through wireless mobile devices, online resources and the Internet access [1, 9]. Many clinicians other than physicians also recognize the importance of scientific evidence in clinical decision making, and evidence-based practice (EBP) is widespread across healthcare disciplines [10].

A systematic review by White et al. [3] found that the use of mobile devices by clinicians is correlated with improved compliance with treatment protocols among patients, as well as improved health outcomes. Access to online health information affect clinicians’ decision-making [5], through improvement in knowledge and changes in patient care decisions [11]. Online professional journals, search engines, i.e., Google and Yahoo, and colleagues are the top information sources used by physicians in diagnosis, treatment and patient care [12]. Other resources often used are UpToDate (80%), Epocrates (46%), Micromedex (36%), Google Scholar (36%) and Cochrane (35%) [3]. Columbia University’s School of Nursing integrated mobile devices into their nurse practitioner curriculum, and informatics competencies for EBP have been incorporated since 2002. The rationale for this was offered by Dr. Bakken when she stated that, *“what clinicians need is decision support tools that fit into their workflow and remind them of evidence-based practice*” [13]. Nursing scholars also have pointed out that EBP is important to nursing practice to increase confidence in decision making and to improve patient outcomes [14, 15].

The most recent survey among clinicians shows that 79% of participants used mobile devices to access clinical information and 72% of participants used clinical applications on their devices [2]. There is substantial evidence of the effectiveness of mobile devices that support decision making in the clinical practice of physicians and educational training programs for medical students. Those studies found that mobile decision support tools, 1) improve compliance to clinical treatment guidelines [16]; 2) increase access to health information at the point of care [17] and in screening, diagnosis, and referrals [18]; 3) improve patient documentation and efficiency [17]; and 4) decrease medical errors [19].

Reliable evidence summaries and validated and expert synthesized resources on smartphone and tablet applications (e.g., UpToDate, DynaMed, or PubMed for Handhelds) can help clinicians’ search for evidence to support the practice of EBM [20]. Point of care decision support tools provide clinicians with useful information in a timely manner for informing patient care [1] and improving patient-clinician communication [21–23]. However, research evidence is lacking on the effectiveness of clinical decision support for clinicians other than physicians. Johnson and colleagues found that the majority of nurse practitioner students perceived that readily available research abstracts at the point of care are useful in informing their clinical decision making, but they cited insufficient time to read the full text of articles as a constraint [15]. Bakken and colleagues studied the effect of mobile decision support on nurse practitioner students to identify health problems, counsel patients, and coordinate care plans. Their randomized controlled trial found higher diagnosis rates and more opportunities for intervention with mobile decision support tools [24].

There are additional barriers to adopting EBM recommendations including time constraints in clinical practice, information overload, restricted access to resources, lack of evidence appraisal skills [25], resources that are not considered trustworthy [15], and/or limited bedside access to online resources [26]. Studies show that when clinicians lack the time to search for answers to clinical questions, they prefer to use their colleagues, supervisors, and/or specialists to answer clinical questions [27–29]. Similarly, barriers that prevent nurse practitioners or nurses from searching for Web-based resources are that they were too time-consuming to access, technically difficult, disrupted the relationship with patients and resulted in longer consultations [30]. Moreover, nurses report difficulty translating and integrating evidence into everyday clinical practice [31]. Therefore, clinicians requested comprehensive resources that answer questions in practice with synthesized evidence [26], protocols, clinical guidelines, clinical decision support tools, prescribing information, or treatment and bottom-line advice [27, 29, 32].

Currently, only two studies [1, 15] tested the usefulness of the Web version of PubMed4Hh among clinicians or nurse practitioner students. Considering the lack of evidence with regard to retrieval of health information that supports clinical decision making among clinicians, our study expands previous research by Fontelo et al. [1] on the usefulness of the PubMed4Hh in clinical practice at the point of care, and make this evidence available to clinicians where it’s needed most. The aim of this study was to demonstrate how evidence provided as abstracts and the bottom-line (TBL) summaries, accessed with mobile devices on a wireless network [PubMed4Hh], were useful for clinicians’ decision-making at the point of care.

## Methods

**Fig. 1:**
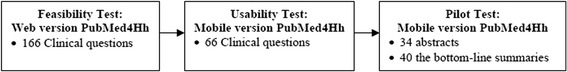
The progression of three phases

This study is a collaborative project between the Lister Hill National Center for Biomedical Communications, National Library Medicine (NLM) and the National Institutes of Health (NIH) Clinical Center Department of Nursing’s Research and Practice Development section. The study was exempted from full Institutional Review Board review by the NIH Office of Human Subjects Research Protections. This pilot study underwent two pre-clinical testing steps which consisted of 1) feasibility testing of a Web version of PubMed4Hh to assess answerability of the clinical questions that are routinely collected by a librarian at the NIH library, and 2) a usability testing of answering clinical questions using PubMed4Hh at point of care by shadowing clinicians during clinical rounds (Fig. 1). The PubMed4Hh original Website underwent re-design based on the results of the two pre-testing phases to improve the interface in the mobile version.

### Feasibility testing of web version PubMed4Hh

A librarian at the NIH Library joined a Pulmonary Branch team and the Pain and Palliative Care Consult service at the NIH Clinical Center to assist finding evidence-based clinical answers that were raised by clinicians during their clinical rounds. The questions were identified by the clinicians and then addressed to the librarian. If the answers were not found at the point of care, the librarian collected the clinical questions, then conducted a search for the answers in her office. The top three answers were then sent to the clinicians. The resources used were: Micromedex, UpToDate, Google, PubMed, Epocrates, or GoodRx on an iPad. One hundred and sixty-six questions were collected from 2009 to 2015 by the librarian who followed the teams during clinical rounds, and saved in Excel. The estimated amount of time to answer each question was recorded. A post-doctoral research fellow used the same 166 questions to search for answers using the Web version of PubMed4Hh in her office. Their results were compared to validate process in the aspect of feasibility testing.

### Usability testing of mobile version PubMed4Hh

The usability testing was an observational study carried out by the post-doctoral research fellow shadowing clinicians during rounds on the following NIH Clinical Center units: Oncology/Hematology, Oncology/Surgical, Pediatrics, Medical and Surgical Telemetry, and Intensive Care for one month from July to August, 2015. Each clinical team conducted patient rounds between 7 am to 10 am, depending on the unit. The post-doctoral research fellow joined patient rounds with PubMed4Hh installed on an iPad. As questions were raised on the status and care plan by the patient’s care team (attendings, fellows, residents, nurse practitioners, nurses, pharmacist and medical students) or by other specialists, the research fellow typed the clinical questions into PubMed4Hh. The search results were evaluated by reading TBL summaries or the abstract, and the most relevant “answers” were selected during clinical rounds. Because of time constraints, only the first few citations were scanned and one or two citations were selected. More citations were reviewed if time allowed before moving on to next patient. Search terms were highlighted in the TBL summary and abstract results for convenience. The process was done independently and results were not shared with clinicians, hence, were not used in the clinical decision-making process. Thus this process did not involve the participation of clinicians. The clinical questions and search results were captured automatically and logged onto a server at the NLM for review. No personally identifiable information on the clinicians, patients or Clinical Center units were captured.

### Pilot testing of mobile application PubMed4Hh

**Fig. 2:**
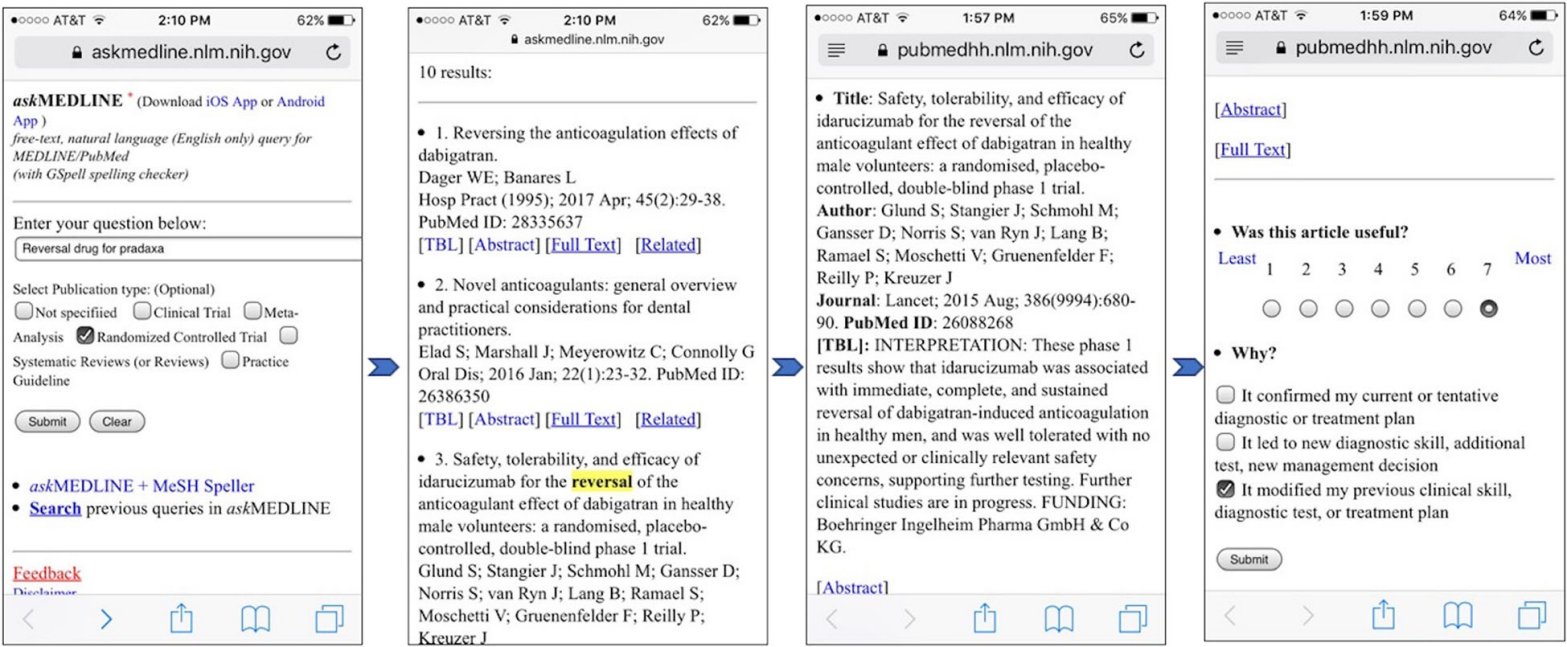
Screen shots of a simulated search example of the re-designed PubMed4Hh mobile Web interface. The first screen shows search term input and types of the studies selected (Randomized controlled trial). The second and third screens show search terms are highlighted in the results. The last screen shows 7-point Likert scale for rating the usefulness of results and rationale for scoring

This pilot study is a non-experimental, descriptive research design via a convenience sampling of clinicians (e.g., physicians, physician assistants, and nurse practitioners) at the NIH Clinical Center from February 2016 to January 2017 replicating the method used by Fontelo et al. [1]. For this study, the PubMed4Hh Website (http://pubmedhh.nlm.nih.gov/hlight2/) [PubMed for Handhelds modified index page] [33] was modified from the original Website (http://pubmedhh.nlm.nih.gov/) [PubMed for Handhelds index page] [34] to include a feature in which key terms are highlighted and an online questionnaire for clinicians to rate the usefulness of abstracts and TBL summaries. Clinicians used the PubMed4Hh search tool using free-text or voice input queries (Fig. 2). The searched questions, summaries read (abstract or TBL summary), and the de-identified rating data were stored in an NLM database. Those data were accessed by the senior investigator and the post-doctoral research fellow.

#### Recruitment of study subjects

Invitations to participate in the study were sent via email to NIH clinicians (physicians, physician assistants, and nurse practitioners) using group email distribution lists, with the option for a one-on-one instruction session for installing the application. Participation was voluntary. Reminders were sent when searches and reviews decreased. A Web-link to an information page for participants was included(https://pubmedhh.nlm.nih.gov/sis) [35]. From the index page, a link to instructions on how to install the application in a mobile phone, how to search clinical questions, and how to rate the usefulness of abstracts or TBLs was provided to participants (https://pubmedhh.nlm.nih.gov/instruction/instruction.html) [36]. Short presentations were done during unit meetings and conferences. Demonstrations were also given to ensure that participants were aware of the search filters, such as, “Meta-Anlysis”, “Systematic Reviews” or “Practice Guideline” (Fig. 2).

#### Online survey questionnaire

Each retrieved citation in the research results page showed both the TBL and abstract and the three evaluation questions (Fig. 2, Additional file 1). The first question was a seven point Likert scale (1 = least useful to 7 = most useful) for users to rate the usefulness of the TBL or abstract while the second question allowed the participants to provide feedback to indicate the rationale for their scores. Those two questions were adopted from Fontelo et al. [1]. The last question asked for the location where they used the application (e.g., clinical rounds, office, or other place). Direct observation of clinicians did not occur, thus the last question’s intent was to clarify where the clinical decision-making occurred (at point of care or elsewere).

#### Data analysis

Descriptive statistics were used to analyze results. The mode and frequency of Likert scores (ordinal) for TBLs and abstracts, and mean and standard deviation (SD) as interval levels were calculated. Independent t-tests were used to compare mean usefulness scores between the TBLs and abstracts. For categorical variables, Chi-square tests were used with continuity correction and ANOVA was used to compare the means scores of usefulness (confirmed, led, and modified). SPSS version 23 (IBM, Armonk, NY) was used for all data analyses.

## Results

### Feasibility testing of web version PubMed4Hh

The amount of time taken by the *librarian* at the point of care to find the right information was similar to the time taken by the post-doctoral research fellow in the office, with a range that generally did not exceed 10 min. Both had a similar result in terms of the number of questions answered: Librarian, 77.1% (*n* = 128), and PubMed4Hh, 77.7% (*n* = 129). More than half of questions (57%, *n* = 94) were answerable by both the librarian using various resources and by the post-doctoral research fellow using PubMed4Hh (Table 1). Only three questions (1.8%) could not be answered by both: 1) “with regard to survival when post-treatment recovery progress is prolonged, is there a survival benefit?”; 2) “what is included in our Clinical Center toxicology screen panel?”; and 3) “what special precautions are needed for laundry with possible VRE contamination?”

**Table 1:** Comparison of clinical question research results by NIH library librarian and a post-doctoral research fellow (*n* = 166 questions)

		Librarian^a^		
		Yes	No	Total
PubMed4Hh used by the post-doctoral research fellow	Yes	94	35	129
	No	34	3	37
	Total	128	38	166

^a^Used Micromedex, UpToDate, Google, PubMed, Epocrates, or GoodRx

Almost 21% of questions (*n* = 34) could not be answered by the post-doctoral research fellow using PubMed4Hh, but were answered by the librarian. Examples of these types of questions were: medications on the hospital’s formulary list; specific information about medication interactions or side effects; and referral or patient transfer information. Similarly, 21% of questions (*n* = 35) were answered by the post-doctoral research fellow using PubMed4Hh, but not by the librarian. The questions that were not answered by the librarian were: risk factors associated with immunosuppressive medications; association between cancer and a specific treatment; cancer prognosis and a specific treatment; or comparisons between medications. On the other hand, some questions required reformulation of questions because relevant results were not found. For instance, the original question searched by the librarian, “what is the mechanism of action in NSAID-induced renal dysfunction” was answered by the post-doctoral research fellow with the modified query, “Nonsteroidal anti-inflammatory drugs and kidney.” More than one-third of questions (39%, *n* = 65) were modified, and most of questions were answerable after modification (94%, *n* = 61) using PubMed4Hh.

### Usability testing of mobile version PubMed4Hh

**Fig. 3:**
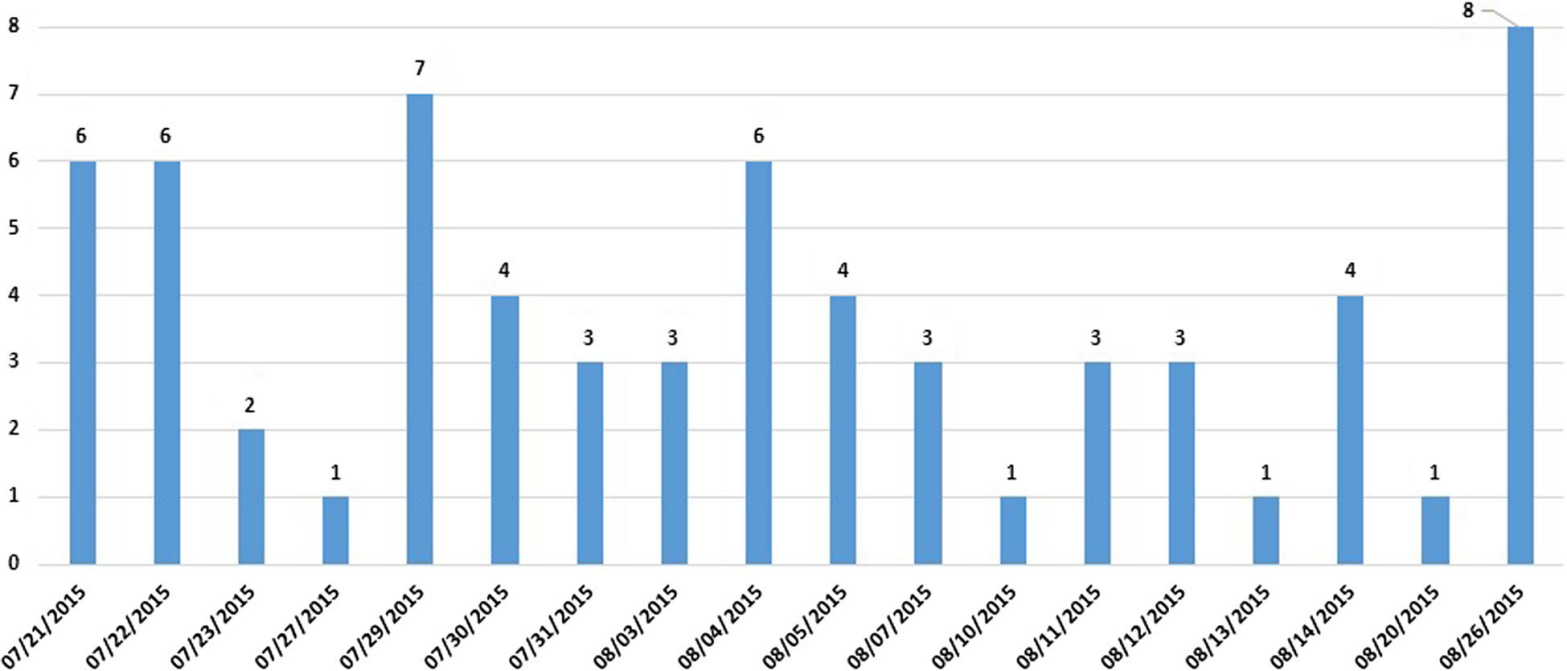
Frequency of number of questions per day

**Fig. 4:**
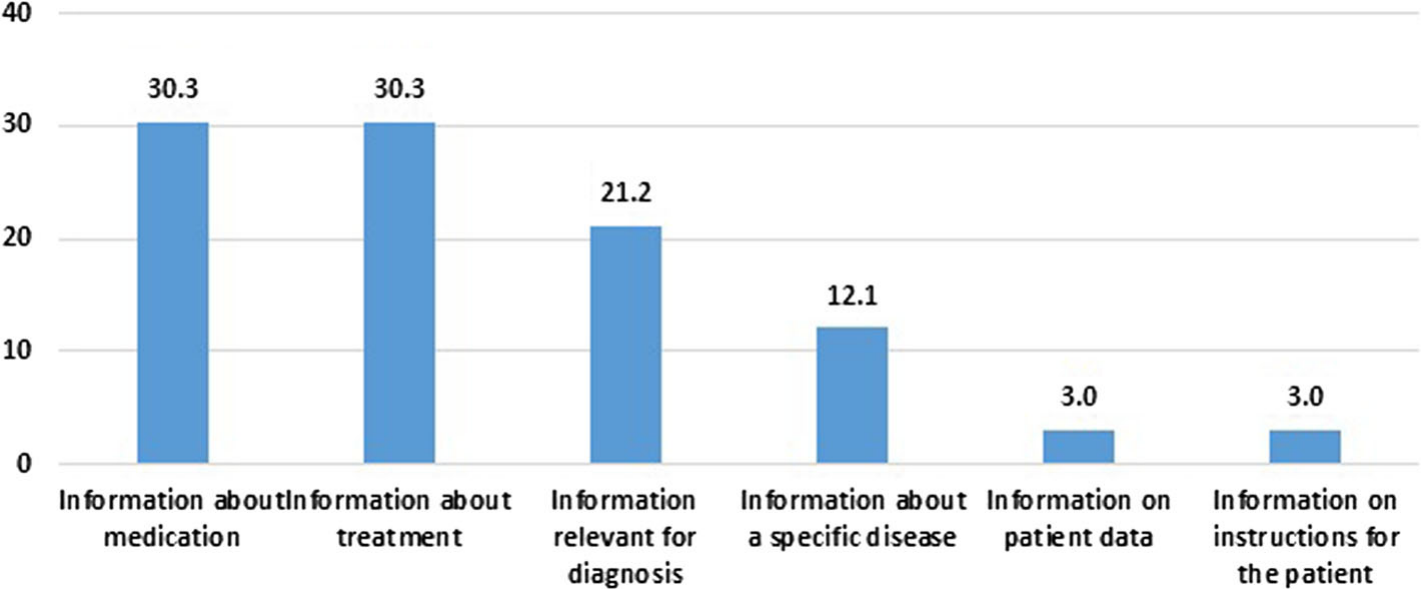
Percentage of questions asked by type

Sixty-six questions were collected during the usability phase. The average number of questions per day was 3.7 (range: 1 to 8, Fig. 3). No specific patterns were observed in the daily questions. The majority of questions collected (90.9%) were generated by physicians and 9.1% were from nurse practitioners. Physicians included residents, fellows, and attending physicians in various specialties. More than three-quarters (77.3%) of questions were from the Intensive Care Unit team, which provided the most clinical care searches compared to other units, followed by Oncology/Hematology/Surgical units (10.6%), Medical/Surgical/Telemetry units (7.5%), and Pediatrics unit (4.5%). Most of the time was dedicated to visits with Intensive Care Units teams which generated more questions compared to the rest of units. More than two-thirds of questions (65.2%) were answerable by the results obtained at the point of care. Almost 35% of questions were not answerable because of lack of time to read the search results, and/or the need to revise the questions when the initial search outcomes were not useable to answer questions. Reading search results and modifying search terms resulted in the resolution of about 26% of the initially unanswerable questions. More than half of questions (60.6%) were related to information about medication or treatment, while 21% were questions pertained to diagnosis, and 12% were specific to disease entities. The least frequently asked questions (6%) were related to information on patient data or information on instructions for the patient (Fig. 4).

### Pilot testing of mobile application PubMed4Hh

Following the usability testing, clinicians who participated in the pilot study reviewed 34 abstracts and 40 TBLs. The number of clinicians who participated in this pilot study is not available because the internet addresses were not collected since participation was voluntary and anonymous. Some participants reviewed one or more TBL summaries or abstracts for some questions. For instance, if they rated both the TBL summary and abstract of a particular journal citation, they were counted as two reviews although only one question was searched. These queries included information about treatment (46%), diagnosis (21%), medication (17%), a specific disease (12%), and on instructions for the patient (4%).

**Fig. 5:**
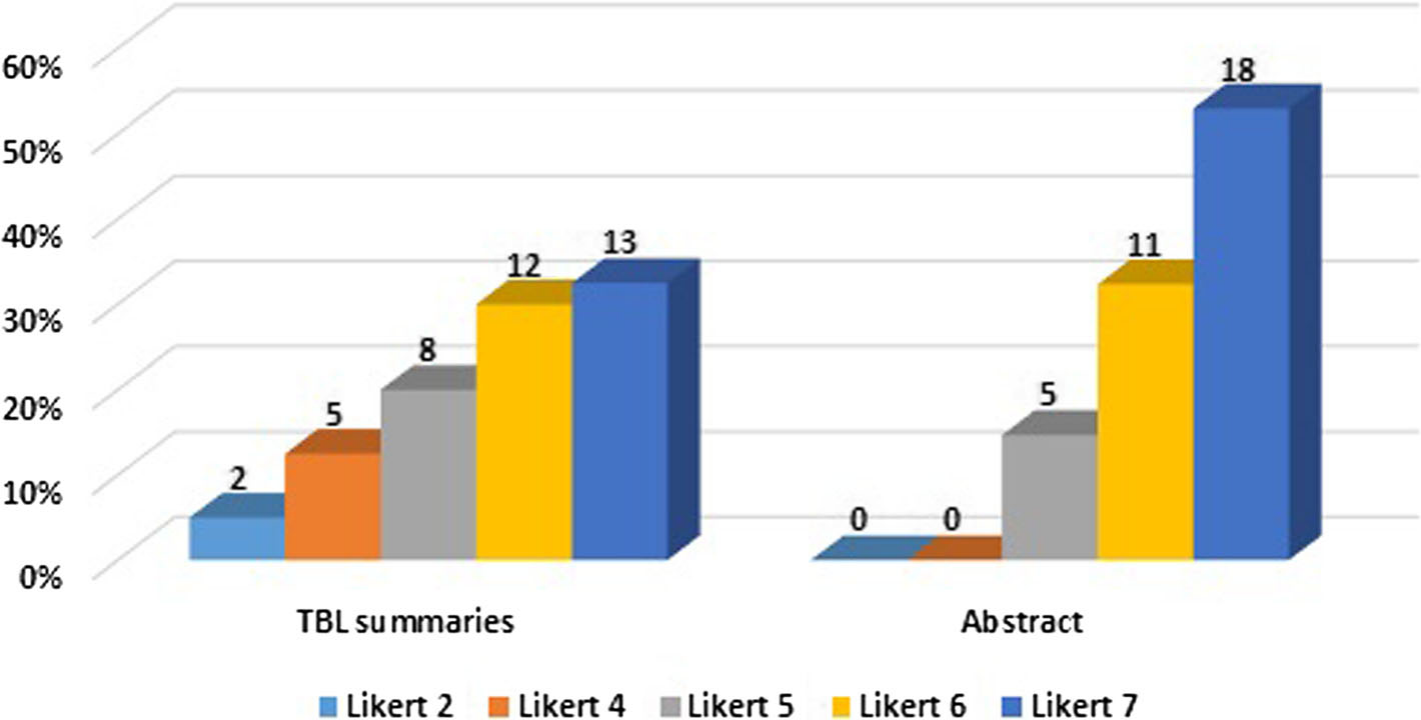
Likert score frequency and percentage: 1 = the least useful, 7 = the most useful. TBL The Bottom-Line

Figure 5 summarizes the usefulness ratings of participants, and 99% (73/74) of scores were based on the review of only one citation. The Likert scale mode for abstracts and TBLs were both ‘7’ (18/34 = 52.9% for abstracts and 13/40 = 32.5% for TBLs). Based on interval estimates, the overall usefulness Likert score for both (1 = the least useful, 7 = the most useful) was 6.00 ± 1.15, 95% CI [5.73, 6.27]. The mean scores for abstracts (6.38 ± 0.74, 95% CI [6.12, 6.64]) was higher than the mean scores for TBL summaries (5.68 ± 1.33, 95% CI [5.25, 6.10]): t (62.76) = − 2.88, *p* = 0.005.

Eighty-nine percent (66/74) of usefulness reviews provided rationales for the Likert score rating. Two participants selected ‘none of the above’ and viewed only TBLs scored both ‘4’ in the usefulness Likert scale. Combinations of reasons for Likert scale scores were also measured (Table 2). ‘Confirmed my current or tentative diagnostic or treatment plan (n=19)’ was combined with ‘all of the above’ (*n* = 22). “None of the above” was treated as missing values. The most frequent single reason (27.3%) was that it confirmed current or tentative diagnostic or treatment plan. There was no difference between abstract and TBLs for providing the rationale of usefulness. Also, there was no statistically significant difference in usefulness scores among three reasons (confirmed, led, and modified).

**Table 2:** Rational for Likert scale scores (*n* = 64)

Rationale	Count	Percentage of total
It confirmed my current or tentative diagnostic or treatment plan	41	27.3
It led to new diagnostic skill, additional test, new management decision	14	21.2
It modified my previous clinical skill, diagnostic test, or treatment plan	9	13.6

Missing (*n* = 10), 13.5%

Choice of ‘other (*n* = 3)’ locations indicated as location of the PubMed4Hh application (e.g., literature review, Metro, and other) was combined with office (*n* = 33). More than half of abstracts or TBLs were used at the office (*n* = 36, 58.9%) compared at the point of care (*n* = 20, 35.7%). The TBLs were used more in the office (*n* = 23, 79.3%), and abstracts were used more at point of care (*n* = 14, 51.9%): *p* = 0.031 (Table 3). There was a statistically significant difference in usefulness mean scores for point of care (6.55 ± 0.76, 95% CI [6.19, 6.91]) versus the office (5.75 ± 1.30, 95% CI [5.31, 6.19]): t (54) = 2.56, *p* = 0.015. However, there was no statistically significant difference in point of care versus office by stratifying two groups: TBLs and abstracts (Table 4).

**Table 3:** Type by location of use (*n* = 56)

Location of use	TBLs n (%)	Abstract n (%)	Chi-square^a^
Point of Care	6 (20.7%)	14 (51.9%)	0.031
Office	23 (79.3%)	13 (48.1%)	
Total	29	27	

*TBL* The Bottom-Line. ^a^Continuity correction. Missing (*n* = 18), 24.3%

**Table 4:** Stratification of abstracts and the TBLs to examine the usefulness score by location (point of care, office) (*n* = 56)

Type	Location	N	Mean (SD)	95% CI	t-test	*p*-value
TBLs	Point of care	6	6.50 (0.837)	5.62, 7.38	1.688	0.103
	Office	23	5.43 (1.472)	4.80, 6.07		
	Total	29	5.66 (1.421)			
Abstract	Point of care	14	6.57 (0.756)	6.13, 7.01	0.980	0.336
	Office	13	6.31 (0.630)	5.93, 6.69		
	Total	27	6.44 (0.698)			

Missing (n = 18), 24.3%

## Discussion

This observational study of practicing clinicians explored the feasibility, usability and pilot testing of the PubMed4Hh application during clinical rounds. The PubMed4Hh interface for mobile devices allowed clinicians to view TBLs and abstracts, and the pilot testing provided evidence of usefulness of both TBLs and abstracts to support clinicians’ clinical decision making at the point of care.

Our study showed similar results to other studies indicating clinicians mostly seek information related to medications, treatments, or diagnoses [8, 12]. A subsequent study by Fontelo et al. [1] also showed similar findings that the majority of questions were related to clinical therapy. Our study outcomes concur with the ‘Evidence Cart study’ by Sackett & Straus [8] that showed that more than three-quarters (81%) of the clinicians’ questions sought evidence about treatment or diagnostic decision (82 and 67% in the usability testing and pilot testing phases, respectively).

The results on the usefulness scores are mostly based on the review of only one citation because the first resource already included the correct answer. The functions of selecting publication types before submitting clinical questions might impact on filtering relevant articles. In addition, the evidence based articles of PubMed4Hh were sorted for relevance, which relied on the most current publications, highlighting key terms in titles, abstracts and TBLs. This study showed the same mode for acceptability of abstracts and TBLs as ‘7’, but showed higher mean scores for both abstracts (6.38) and TBLs (5.68) compared to an earlier study [1], where the scores were 4.77 for abstracts and 5.09 for TBLs. Abstracts were slightly preferred over TBL summaries by 1.4:1, which may be unique to the types of clinicians at the NIH Clinical Center, who may prefer more “detailed” information to confirm their practice for research patients. Fontelo et al. [37] shows the similar findings that structured abstracts are informative and useful to clinicians as a resource for guiding clinical decisions. Meanwhile, the earlier study explained their TBL preference was due to the easy-to-read essential information that provide a quick evidence source [1]. This was also suggested by the ‘Evidence Cart’ in 1998 [8]. These conflicting findings may need further investigation with a larger population of clinicians. Another possible explanation may be the different types of devices using the application, and its readability on the device.

This study seems to confirm further that in the majority of instances, evidence in TBLs and abstracts may affect clinical decisions making. ‘Confirmed my current or tentative diagnostic or treatment plan’ accounted for the primary rationale for usefulness (27%), followed by ‘led to new diagnostic skill, additional test, new management decision’ (21%) and ‘modified my previous clinical skill, diagnostic test, or treatment plan’ (14%). Although 18 years after the practice of using evidence-based literature in Evidence Carts, the opinion of clinicians on the usefulness of evidence in their practice seems to be similar, whether they use a physical cart carried around during rounds or mobile applications through wireless devices (“virtual evidence cart”). However, the earlier study of the Web version of PubMed4Hh [1] found a slightly different rationale when clinicians made decisions: 1) led to new diagnostic skill, additional test, new management decision (44%), 2) confirmed my current or tentative diagnostic or treatment plan (29%), and 3) modified my previous clinical skill, diagnostic test, or treatment plan (27%). In that study, the difference was explained by the availability of more tests and treatment options accessing the Internet, leading to the selection of ‘led to new diagnostic skill, additional test, and new management decision’. The population studied was also different since it involved clinicians worldwide. Based on the observation during usability testing to shadow clinicians, the clinical discussion on questions were more likely based on the physician’s recall of knowledge from textbooks, research protocols, and literature. Consequently, in clinical research settings such as the NIH Clinical Center (all patients are participants in a clinical trial), clinicians rely heavily on research protocols to make clinical decisions. This may explain why the top rationale in our study was “confirmed my current or tentative diagnostics or treatment plan”, which is different than previous research findings in non-research hospitals. Because an earlier paper [1] concluded that evidence based clinical decision-making shifted from confirming current or tentative practice to leading clinicians to newer patient engagement skills, these findings should be cautiously interpreted and re-validated in heterogeneous clinical settings to confirm the generalizability.

We asked whether the evidence based literature searches were conducted with clinical questions at the point of care (i.e., clinical rounds, bedsides) or not (i.e., office or other places). We found that the PubMed4Hh mobile application was more often used in office compared to the point of care. Interestingly, the TBLs were used more often at the office, not at the point of care. However, the total number of used at the office (36) was more than usage at the point of care (20). Based on the observations during usability testing, there are many reasons why clinicians may not use technology to research clinical questions at the point of care, lack of time during clinical rounds being a major factor [2]. A nurse practitioner commented that there was simply no time to search clinical questions and read the information retrieved at the point of care. Although more than half of clinicians preferred to investigate their clinical questions right away [2], some clinicians in this study reported that they preferred spending more time to find evidence in office after clinical rounds to confirm their clinical diagnosis or management. Another factor is that they may not be comfortable looking up evidence in front of the team during clinical rounds and would prefer to do so after rounds, or they may feel it interferes with their workflow. Or, certain clinicians may be sensitive to the fact that others may think they are using their device for personal reasons rather than work reasons if they are using it during rounds to look up evidence. This study, however, seems to demonstrate that it is feasible to find evidence that may be useful for making clinical decisions at the point of care. The resource that we used in this study is PubMed4Hh, but there are many others, both free and subscription-based. TBL was preferred from the earlier study [1], because they provided easy-to-read summaries that captured the essential information from the literature. However, the original study did not assess the usefulness of TBL at the point of care. Brassil et al. [2] found that clinicians have no formal training in searching databases or they may not be aware of available digital resources, may explain the preference for TBLs. Due to time constraints during rounds, the search on PubMed could be deemed too broad and the results retrieved irrelevant, or if too specific, may not provide the needed results in time for decision making. This type of search could be instead be more useful if done in the office, when more time is available to go into the details of the clinical question. For these clinicians, PubMed4Hh may be a useful resource to use to support their decision making at the point of care with TBL summary feature that integrates their clinical work flow.

Usability testing of the PubMed4Hh revealed areas that needed to be improved related to the tool’s interface and content, in addition to identifying relevant biomedical literature in a short time period at the point of care. Our iterative process of testing the application and content revision within the mobile interface allowed us to successfully pilot test with clinicians at the point of care. Clinicians searched for more evidence at the office compared than at the point of care. Other studies found that clinicians who use evidence-based guidelines to make treatment decisions and care plans were more likely to identify health issues in patients using mobile application [13]. Johnson et al. [15] found that nurse practitioner students perceived the usefulness of the research abstracts for their clinical decision-making using the Web version of PubMed search. Similar to observations of the PubMed4Hh usability testing by shadowing clinicians at the point of care, pilot testing revealed workflow challenges because it did not support our assumption that it would be more convenient to use TBLs compared to use of abstracts at the point of care. However, our study results agree with the suggestions from the Johnson et al. [15] that providing readily available access to research abstracts at the point of care is useful for clinicians. Replication and adaption of findings in this study requires further validation. A next step could be the integration in the clinical workflow [15] and assigning a member of the rounding team to search for evidence.

Finally, we learned a lesson on participant recruitment: we got more responses when a clinician who practiced in NIH Clinical Center sent the email on behalf of the post-doctoral research fellow. Additionally, when clinical nurse specialists introduced the post-doctoral research fellow to clinicians, they seemed to be more attentive to the presentation and demonstrations at the meeting. As McFadyen and Rankin [38] suggest, involving a gatekeeper early in research seemed to benefit recruiting participants because it resolved communication issues. This observation seems to be validated by our own experience.

## Limitations

This study was conducted in a research hospital, which limits the generalizability of study findings to other non-academic settings. However, the need for evidence to inform clinical practice is universal so the results may still apply in other settings. Additionally, the evaluation of the search results during the feasibility testing phase was done by one person, thus Kappa consensus was not calculated. It may bias the data on answerability of the questions. Second, during the usability testing phase, research was not done by the clinicians themselves who may have had more knowledge of the patient’s clinical condition. This presents subjective limitations including the identification of the subject or topic that would need to be searched, but also introduces a valid assumption that it is almost certain the researcher would not use the same search terms as the actual clinician subjects (e.g., variability in search terms among users) [39]. Third, the highlighting of search terms in the search results during the pilot testing phase seems an insufficient measure of the value and accuracy of the search result and/or the content itself which was not our primary intention to measure. Fourth, the categorization of the questions is subjective (e.g., medication, diagnosis, treatment, specific disease, patient data, or instructions for the patient), so that the accuracy and validity should be carefully interpreted. Fifth, the generalizability of results should be cautiously viewed due to the small sample size. However, this pilot study was the first step in testing the usefulness of the PubMed4Hh mobile application for clinical decision making at the point of care and finding ways to incorporate it into clinicians’ workflow. Sixth, the participants in the pilot study may be more technologically savvy than non-participants and may not represent general clinicians. Seventh, response biases may exist because of the self-administered Web survey. For example, clinicians who were interested in the topic of clinical decision making at point of care may be more likely to respond. Therefore, the results should be interpreted with caution. Eighth, PubMed4Hh has no algorithm to suggest reformulation of search questions when search results were not successful during clinical rounds, but in future it may be worthwhile to investigate how automated suggestions to modify search questions might be helpful to clinicians. Finally, this pilot study did not collect the personal information of the participants that may have important information to analyze by the discipline of the participants, type of their practices, size of the institutes in which they practice, age, gender, etc. Future studies might need to address many of these limitations and expand this study to the bigger and heterogeneous population of point of care clinicians.

## Conclusion

The feasibility of using the application during rounds was confirmed by the amount of time taken to find the right information at office was similar to the time taken by the librarian at point of care. The usability testing showed that real-time search and retrieval of reliable and potential useful information for clinical decision making is feasible at the point of care. Our pilot study results showed that retrieving relevant health information from biomedical literature using PubMed4Hh is useful for decision making at the point of care as well as office. In this study with clinicians, information access at the office was higher (79.3%) than at the point of care. Abstracts were used more often but not significantly over TBLs and no significant difference was found in the rationale for scoring the evidence (confirmed, led, and modified), although participants seemed to favor “confirm” more. However, there was significant difference in use of abstracts versus TBLs based on location. The impact of this PubMed4Hh mobile application could be significant, introducing the application in education for medical and nursing students, and which might be the most significant contribution of this study.

## Additional file

Additional file 1: Online Survey Questionnaire, Online questionnaire for clinicians to rate the usefulness of abstracts and TBL summaries. Also shown on the right panel in Fig. 2. (DOCX 51 kb)

## Abbreviations

EBM: Evidence-based Medicine
EBP: Evidence-based Practice
NIH: National Institutes of Health
NLM: National Library Medicine
PubMed4Hh: PubMed for Handhelds
TBL: The Bottom-Line
