# Supplementary material for: Clinicians’ perceptions of usefulness of the PubMed4Hh mobile device application for clinical decision making at the point of care: a pilot study
Source: BMC Med Inform Decis Mak. 2018 May 8;18:27. doi: 10.1186/s12911-018-0607-9 (PMC5941474; doi:10.1186/s12911-018-0607-9)
Supplement: Supplementary file 1 — Online Survey Questionnaire, Online questionnaire for clinicians to rate the usefulness of abstracts and TBL summaries. Also shown on the right panel in Fig. 2. (DOCX 51 kb) [file 12911_2018_607_MOESM1_ESM.docx]

**Online Survey Questionnaire**

Description of data: Online questionnaire for clinicians to rate the usefulness of abstracts and TBL summaries. Also shown on the right panel in Fig. 2

**Q1. Useful for clinical decision making?**

Least  1 2 3 4 5 6 7 Most

**Q2. I rated this TBL/Abstract based on one or more of the following:** (Choose one or more)

    It confirmed my current or tentative diagnostic or treatment plan

    It led to new diagnostic skill, additional test, new management decision

    It modified my previous clinical skill, diagnostic test, or treatment plan

    All of the above

    None of the above

**Q3. When did you use the TBL/Abstract for clinical decision making?**

    At point of care (e.g., clinical rounds, bedside rounds)

    At my office

    Other, please specify
